# Mapping evidence of spinal manipulation therapy for headaches in South Africa: a scoping review of grey literature

**DOI:** 10.1186/s12906-025-05044-0

**Published:** 2025-08-01

**Authors:** Keseri Padayachy, Ismail Fatima, Morris Kahere, Alister du Rose, Katherine A. Pohlman

**Affiliations:** 1 Department of Chiropractic, Durban University of Technology, Durban, South Africa; 2 Department of Chiropractic, Faculty of Health Sciences, University of Johannesburg, Doornfontein, South Africa; 3 Reliable Spine, Scoliosis and Spine Pain Care, Coylton, Ayr, Scotland, United Kingdom; 4 Health Sciences University, AECC School of Chiropractic, Bournemouth, England; 5 Research Center, Parker University, Dallas, TX USA

**Keywords:** Chiropractic, Headache, Outcome measures, Grey literature, South Africa

## Abstract

**Objective:**

Spinal manipulative therapy (SMT) has been demonstrated to be an effective management approach for primary headaches; however, current literature often excludes data from South Africa (SA). The use of grey literature provides a viable mechanism to address knowledge gaps. Understanding that Master’s dissertations are a source of grey literature, this review’s primary objective was to address the following question: What is the range of evidence, particularly regarding subjective and objective outcome measures, for the application of SMT in managing headache patients at chiropractic training facilities in SA?

**Methods:**

A scoping review methodology was adopted in compliance with the Joana-Briggs-Institute and the Arksey and O’Malley frameworks and reported following the preferred reporting items for systematic reviews and meta-analysis extended for scoping reviews checklist. The search was performed using the Durban University of Technology and University of Johannesburg Research Databases. All studies conducted from 1995 to May 2023 were retrieved. Trials conducted with SMT for the management of headaches were included and subjective (i.e., numerical rating scale, headache disability index, neck disability index) and objective (i.e., range of motions, pressure algometry) outcomes were extracted.

**Results:**

In total 25 dissertations with 921 headache patients were reviewed. Across most of the dissertations, combining SMT with additional modalities versus SMT alone or another modality alone yielded greater improvement in subjective outcome measures, although there were occasional exceptions where no clear pattern emerged. In terms of objective measures, there were both increases and decreases across the different interventions.

**Discussion:**

The findings align with existing literature, indicating that primary headache patients in SA who receive SMT in conjunction with other non-pharmacological treatments respond favourably. This study underscores the potential value of grey literature, particularly in regions where high-quality data is scarce. It highlights the significance of SMT for policymakers, funders, and other stakeholders involved in managing headache patients in SA. Although limitations related to the quality of the dataset are acknowledged, the standardization and robust design of clinical trial protocols at SA institutions reveal numerous strengths. Despite ongoing discussions in the literature regarding the use of SMT for headache management, there is a strong case for existing literature to be used in the SA context.

**Clinical trial number:**

Not applicable.

## Introduction

The global prevalence of headache disorders imposes a substantial economic burden on healthcare systems [1]. Although there are fewer publications from the sub-Saharan Africa region compared to high-income (HI) regions, headache disorders remain a public health priority in the sub-Saharan Africa region, including South Africa (SA) [2, 3]. Primary headache disorders, including migraines and tension-type headaches, where there is an absence of an underlying pathologic process [4], remain infrequently identified and insufficiently managed in these populations, with a notable lack of population-specific epidemiological data [5]. However, several barriers hinder effective care provision in this region. These barriers include a shortage of specialists capable of accurate diagnosis, high rates of medication overuse, political and economic obstacles to care delivery, and social factors such as community misconceptions about headache conditions [6]. Specifically in SA, the National Development Plan and health policy seek to reduce the burden of non-communicable diseases and improve health outcomes. Yet, there is minimal investment in this context [7], and African countries are frequently excluded from clinical trials in this area likely due to lack of funding [8].

In the management of headache disorders, spinal manipulative therapy (SMT) is commonly considered by patients [9–12] and is recommended in numerous guidelines for headaches and migraines [9, 13–17]. Indeed, a recent systematic review exploring the effectiveness of manual therapies in the treatment of cervicogenic headache, concluded that SMT can improve associated symptoms, and that adding SMT in combination with other modalities can assist in maintaining longer term results [18]. However, different countries, particularly in Africa, have unique cultural practices, lifestyles, and environmental conditions that can influence headache diagnosis, management and treatment options [19]. While evidence-based guidelines incorporate regional data to ensure tailored recommendations for better treatment outcomes, in sub-Saharan nations like SA there remains a gap in research that addresses the regional and contextual factors influencing SMTs effectiveness and accessibility for headache management [7]. This gap is particularly evident in underserved communities, where healthcare disparities, cultural diversity, and limited access to specialized care affect headache management outcomes [7]. This raises concerns about what constitutes as effective treatment of headache disorders for such regions. Given the limited funds and data availability– compounded by competing healthcare priorities - it is essential to identify solutions that can significantly benefit headache patients and optimize the existing health systems in these regions.

One solution to the lack of data is the use of ‘Grey Literature.’ Grey literature is an umbrella term for information produced outside of traditional publishing mechanisms and encompasses academic papers, post-graduate theses, and dissertations [20, 21]. Despite some obvious limitations, such as variation in quality and the absence of a recognised peer review process, grey literature is considered a potentially valuable resource with more up-to-date data [20, 22].

For the past two decades, two chiropractic teaching programmes in SA have been conducting research for Master’s dissertations, including studies on the clinical management of headache disorders. At these institutions, research prioritizes the safety of both participants and researchers by adhering to established ethical standards and research integrity, overseen by their respective research ethics committees. These committees are responsible for conducting ethical review and granting clearance for all research activities. Additionally, both institutions maintain active registration and collaboration with the SA National Health Research Ethics Council [23].

Recognizing that data from Master’s dissertations can serve as a crucial source of evidence-based knowledge to address the current data gap in headache management in SA, this review aims to examine dissertations from two SA chiropractic teaching programs. Specifically, it seeks to answer the following question: What is the scope of evidence, specifically subjective and objective outcome measures, for the use of SMT provided at chiropractic training facilities, in the management of primary headache patients in SA?

## Methods

We adopted a scoping review methodology, which was deemed appropriate to answer the research question. This review was conducted in accordance with the Joana-Briggs-Institute [23] and the Arksey and O’Malley [24] frameworks for scoping reviews, and it was reported following the Preferred Reporting Items for Systematic Reviews and Meta-analysis extension for Scoping Reviews (PRISMA-ScR) checklist [25]. No review protocol exists for this study. The review involved the following five steps: (i) identification of the research question, (ii) identification of relevant studies, (iii) selection of eligible studies, (iv) charting the data, and (v) collating and summarising the data.

### Identification of the research question

This review aimed to answer the research question: “What is the scope of evidence, specifically subjective and objective outcome measures, for the use of SMT provided at chiropractic training facilities, in the management of primary headache patients in SA?”. The population-concept-context framework, outlined in Table 1, was used to set the eligibility of the research question following recommendations from the Joana-Briggs-Institute [26]. Studies were included if they were Masters dissertations conducted by post-graduate chiropractic students at the two universities in SA, the Durban University of Technology (DUT) or the University of Johannesburg (UJ). Only clinical trials investigating chiropractic spinal manipulation in the management of primary headache patients with the following designs: Comparative (randomised with at least one of the comparison groups including SMT), Experimental (randomised with the comparison group NOT including SMT), Observational (quasi-experimental with at least one group including SMT) were included. Headache studies that were outside the context of SA were excluded.

**Table 1:** The population-concept-context framework for study eligibility

Criteria	Determinant
Population/Participants	Individuals/patients presenting (with)/experiencing primary headaches of any type, severity, or duration, including but not limited to tension-type headaches, migraine headaches, cluster headaches, and cervicogenic headache across age groups above 18 years, across all demographic characteristics.
Concept	Intervention: Chiropractic spinal manipulation therapy (SMT) of the cervical spine/other musculoskeletal structures to alleviate pain, improve function, and promote overall health, performed by Masters chiropractic students under the supervision of chiropractic clinicians. Study Design: Comparative (randomised with at least one of the comparison groups including spinal manipulation therapy), Experimental (randomised with the comparison group NOT including spinal manipulation therapy), Observational (quasi-experimental with at least one group including spinal manipulation therapy).
Context	The studies included in this review were dissertation projects conducted in South Africa by Masters-level chiropractic students at either University of Johannesburg (UJ) or Durban University of Technology (DUT). Their dissertations, in partial fulfilment of their qualification requirements, underwent an internal peer-review process during the marking stage to ensure rigor and quality.

### Search strategy for the identification of relevant studies

**Fig. 1:**
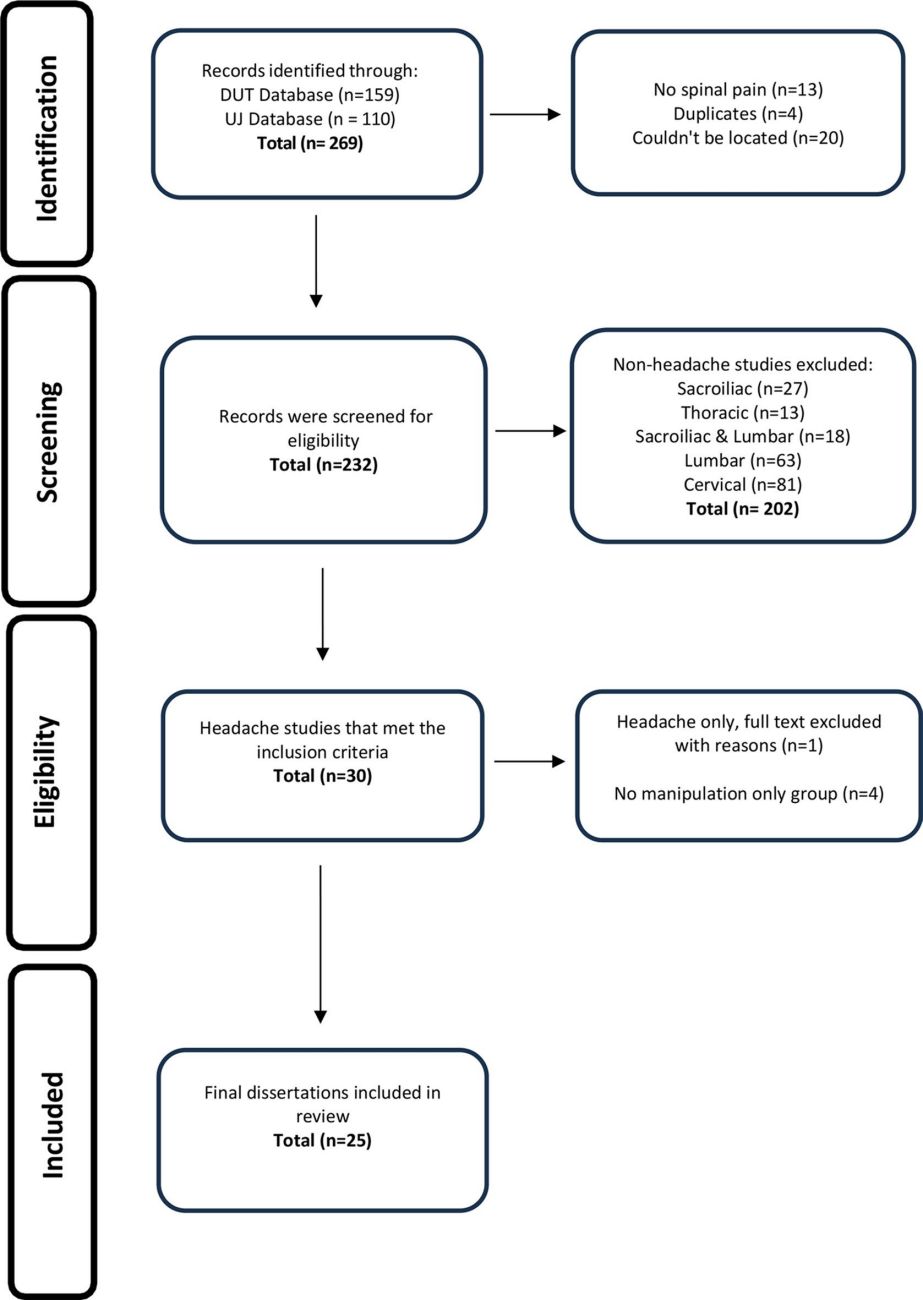
PRISM-ScR flow chart of dissertation databases at DUT and UJ

As described in the Cochrane Handbook for Systematic Reviews [26] and further detailed by Godin et al. 2015 [22], a grey literature search plan must be developed *a priori.* Given that this study focused on dissertations from chiropractic programs in SA, a search strategy was tailored specifically to each university’s thesis database. The date range was set from January 1995 for DUT and 2005 for UJ - the years when their first dissertations were published- up to May 2023, when the scoping review commenced. As shown in Fig. 1, all titles were retrieved directly from the respective institution’s records.

### Selection of eligible studies

All studies retrieved from the institutional databases search were exported to Google Sheets (Google LLC. Menlo Park, California, United States of America) for initial screening by five independent reviewers (MK, KAP, KP, AD and FI) to identify eligible titles. Any disagreements among the reviewers were resolved by full-team discussion. The PRISMA-ScR (Fig. 1) illustrates the review process. Studies were included if they were conducted by chiropractic students at DUT and UJ in SA and provided SMT to the cervical spine in isolation or in combination with other modalities for the management of headaches. Only prospective studies that were either comparative, experimental, or observational were included.

### Charting the data

Data from all included studies were extracted using a data extraction form that was developed and pilot tested a priori for reliability and consistency in data collection by the reviewers. Necessary amendments were made prior to the final use of the data collection tool. The following information were extracted: author/student, site (DUT or UJ), study completion year, study design, condition, number of active treatment visits and time span, total sample size, study groups with sample sizes, subjective baseline outcome measures and objective outcome measures. Both subjective (patient perspective) and objective (quantifiable data) were extracted to provide a well-rounded assessment of intervention efficacy, ensuring that both patient perspectives and quantifiable outcomes are captured [27, 28]. For the outcome measures included, the mean change was determined from the available data in the manuscript if not directly provided. Although the initial intent was to include other descriptive measures, such as standard deviations for calculating confidence intervals, these data were not consistently available. The extracted data were then collated, analysed, and summarised in Table 2.

**Table 2:** Details of studies included in this scoping review (*n* = 25)

Student	Site*	Year	Study Design^	Condition	Number of active treatment visits and timespan	Total Sample (*n*)	Groups (*n*)	Subjective Outcome Measures	Objective Outcome Measures
Da Silva, KL [59]	^a^ DUT	1994	Comparative- Randomised clinical trials	Muscular tension-type headache	2 visits over 5 weeks	30	1) Manipulation (*n* = 15) 2) Manipulation and music therapy (*n* = 15)	a- Headache Questionnaire b- Neck Disability Index Symptom Diagram	d- Cervical Range of Motion Instrument
Angus, AK [58]	DUT	1997	Comparative- Randomised clinical trials	Tension-type headache	10 visits over 4 weeks or until clinically asymptomatic	30	1) Manipulation (*n* = 15) 2) Manipulation and cryotherapy (*n* = 15)	b- Neck Disability Index c- Numeric Pain Rating Scale (0-100 scale) McGill Pain Questionnaire-Short Form Cervical ROM Impairment Rating	^c^ NA
Cullinan, A [57]	DUT	1998	Comparative - Randomised clinical trials	Migraine headache	10 visits over 4 weeks	30	1) Manipulation (*n* = 15) 2) Manipulation and acupuncture (*n* = 15)	b- Neck Disability Index McGill Pain Questionnaire-Short Form	d- Cervical Range of Motion Instrument
Donkin, R [60]	DUT	1998	Comparative- Randomised clinical trials	Tension-type headache	9 visits over 4 weeks	30	1) Manipulation (*n* = 15) 2) Manipulation and manual traction (*n* = 15)	b- Neck Disability Index c- Numeric Pain Rating Scale (0-100 scale) McGill Pain Questionnaire-Short Form Headache Diary	d- Cervical Range of Motion Instrument
Thomson, DA [56]	DUT	2000	Experimental- Randomised clinical trials	Tension-type headache	2 visits over 48 h	70	1) Manipulation- cervical spine (*n* = 35) 2) Acetaminophen 1000 mg / Caffeine 130 mg (*n* = 37)	b- Neck Disability Index c- Numeric Pain Rating Scale (0-100 scale) McGill Pain Questionnaire-Short Form	d- Cervical Range of Motion Instrument e- Pressure Pain Threshold
Kidson, MAR [54]	DUT	2001	Experimental - Randomised clinical trials	Episodic tension-type headaches	2 visits over 2 weeks	60	1) Manipulation- cervical spine (*n* = 30) 2) Acetaminophen 500 mg (*n* = 30)	a- Neck Disability Index c- Numeric Pain Rating Scale (0-100 scale) McGill Pain Questionnaire Headache Diary	e- Pressure Pain Threshold
Cartwright, GD [55]	DUT	2002	Comparative- Randomised clinical trials	Chronic tension-type headache	9 visits over 2 weeks	30	1) Manipulation-cervical spine (*n* = 15) 2) Manipulation-cervical spine and nocturnal bite guard (*n* = 15)	b- Neck Disability Index c- Numeric Pain Rating Scale McGill Pain Questionnaire	d- Cervical Range of Motion Instrument
Fonseca, SW [53]	DUT	2002	Comparative- Randomised clinical trials	Chronic tension-type headache	4 visits over 17 days	30	1) Manipulation- cervical spine (*n* = 15) 2) Manipulation and placebo TENS (*n* = 15)	McGill Pain Questionnaire-Short Form Headache Diary	NA
Prithipal, A [52]	DUT	2003	Comparative- Randomised clinical trials	Episodic tension-type headache	5 visits over 2 weeks	60	1) Manipulation- cervical spine (*n* = 20) 2) Interferential current therapy (*n* = 20) 3) Combination of Groups 1 & 2 (*n* = 20)	c- Numeric Pain Rating Scale (0-100 scale) Headache Diary	e- Pressure Pain Threshold Myofascial Diagnostic Scale
du Preez, L [51]	DUT	2004	Comparative- Randomised clinical trials	Migraine headache	8 visits over 6 weeks	30	1) Manipulative- cervical spine (*n* = 10) 2) Homeopathic migraine complex pills (*n* = 10) 3) Combination of Groups 1 & 2 (*n* = 10)	b- Neck Disability Index Glasgow Homeopathic Hospital Outcome Score	NA
Legoete, K [50]	DUT	2010	Experimental - Randomised clinical trials	Episodic tension-type headache	5 visits over 4 weeks	32	1) Manipulation- cervical spine (*n* = 16) 2) Ibuprofen^®^ taken daily for 7 days (*n* = 16)	b- Neck Disability Index c- Numeric Pain Rating Scale (0-100 scale) Headache Diary McGill Pain Questionnaire-Short Form	NA
Trollope, LJW [49]	DUT	2010	Experimental - Randomised clinical trials	Episodic tension-type headache	6 visits over 4 weeks	45	1) Manipulation- cervical spine (*n* = 15) 2) Dry needling (*n* = 15) 3) Combination of Group 1 & 2 (*n* = 15)	a- Headache Disability Index Headache Diary	d- Cervical Range of Motion Instrument e- Pressure Pain Threshold
Judelman, N [36]	^b^ UJ	2011	Comparative- Randomised clinical trials	Cervicogenic headache	6 visits over 3 weeks	48	1) Manipulation- cervical spine (*n* = 16) 2) Myofascial dry needling therapy (*n* = 16) 3) Combination of Group 1 & 2 (*n* = 16)	a- Headache Disability Index b- Neck Disability Index c- Triple Visual Analogue Scale - standardized to a Numeric Pain Rating Scale	d- Cervical Range of Motion Instrument
Workman, SJ [46]	UJ	2011	Observational	Chronic cervicogenic headaches	7 visits over 3 weeks	30	1) Manipulation (*n* = 30)	a- Headache Disability Index b- Neck Disability Index c- Numeric Pain Rating Scale	d- Cervical Range of Motion Instrument
Keshav, T [39]	UJ	2012	Comparative- Randomised clinical trials	Cervicogenic headache	6 visits over 3 weeks	30	1) Manipulation- upper cervical spine (*n* = 15) 2) Manipulation- upper cervical spine with interferential current and ultrasound therapy (*n* = 15)	a- Headache Disability Index c- Numerical Pain Rating Scale	e- Pressure Pain Threshold
Orkan, S [43]	UJ	2012	Experimental - Randomised clinical trials	Tension-type headache	6 visits over 3 weeks	32	1) Manipulation- cervical spine (*n* = 16) 2) Acupuncture points needling (*n* = 16)	a- Headache Disability Index b- Neck Disability Index c- Numeric Pain Rating Scale	d- Cervical Range of Motion Instrument
Moosajee, N [40]	UJ	2013	Comparative - Randomised clinical trials	Tension-type headache	6 visits over 2 weeks	48	1) Manipulation- temporomandibular joint (TMJ) (*n* = 16) 2) Ischemic compression- lateral pterygoid muscle (*n* = 16) 3) Combination of Group 1 & 2 (*n* = 16)	a- Headache Disability Index TMJ Symptom Questionnarie	Temporomandibular Joint motion - Vernier Calipers
Chopdat, SH [37]	UJ	2015	Observational	Classical migraine (migraines with aura)	5 visits over 4 weeks	20	1) Manipulation- cervical spine (*n* = 20)	a- Headache Disability Index - MIDAS (Migraine Disability Assessment Test) Headache Diary	NA
Newman, P [42]	UJ	2015	Comparative- Randomised clinical trials	Tensioin-type headache with forward head posture	6 visits over 3 weeks	30	1) Manipulation- cervical spine (*n* = 10) 2) Soft tissue protocol (*n* = 10) 3) Combination of Group 1 & 2 (*n* = 10)	a- Headache Disability Index b- Neck Disability Index	e- Pressure Pain Threshold
Omar, S [41]	UJ	2015	Comparative - Randomised clinical trials	Tension-type headache	6 visits over 2 weeks	30	1) Manipulation- cervical spine (*n* = 10) 2) Low-level laser (*n* = 10) 3) Combination of Groups 1 & 2 (*n* = 10)	c- Numeric Pain Rating Scale Headache Impact Test (HIT-6)	e- Pressure Pain Threshold
Seejarim, T [45]	UJ	2016	Comparative- Randomised clinical trials	Tension-type headache	6 visits over 3 weeks	30	1) Manipulation- cervical spine (*n* = 10) 2) Muscle energy technique (*n* = 10) 3) Combination of Groups 1 & 2 (*n* = 10)	c- Numeric Pain Rating Scale Headache Impact Questionnaire	d- Cervical Range of Motion Instrument e- Pressure Pain Threshold
Dulabh, K [38]	UJ	2017	Comparative- Randomised clinical trials	Cervicogenic headache	6 visits over 3 weeks	30	1) Manipulation- cervical spine (*n* = 21) 2) Electromechanical adjusting instrument (*n* = 20)	a- Headache Disability Index c- Numerical Pain Rating Scale	d- Cervical Range of Motion Instrument
Orr, CR [44]	UJ	2018	Comparative - Randomised clinical trials	Tension-type headache	4 visits over 2 weeks	30	1) Manipulation- cervical spine (*n* = 10) 2) Muscle tension release technique (*n* = 10) 3) Combination of Groups 1 & 2 (*n* = 10)	b- Neck Disability Index c- Numeric Pain Rating Scale Headache Impact Questionnaire	e- Pressure Pain Threshold
Whittaker, R [48]	DUT	2018	Experimental - Randomised clinical trials	Cervicogenic headache	6 visits over 3 weeks	41	1) Manipulation- cervical spine (*n* = 21) 2) Electromechanical adjusting instrument (*n* = 20)	a- Headache Disability Index b- Neck Disability Index c- Numerical Pain Rating Scale	d- Cervical Range of Motion Instrument
Brann, WE [47]	DUT	2020	Experimental - randomised controlled trials	Cervicogenic headache	1 visit	45	1) Manipulation- cervical spine (*n* = 15) 2) Placebo (*n* = 15) 3) Control (*n* = 15)	a- Headache Disability Index c- Numeric Pain Rating Pain Scale Headache Diary	Surface Electromyography Biopac-TSD121C dynamometer

^ - Comparative (randomized with at least one of the comparison groups including spinal manipulation), Experimental (randomized with the comparison group NOT including spinal manipulation), Observational (quasi-experimental with at least one group including spinal manipulation) were included

^a^ DUT: Durban University of Technology

^b^ UJ: University of Johannesburg

^c^ NA: Not Applicable

### Collating and summarising the data

The extracted data were continually reviewed to improve the quality of the collated and summarized evidence. The authors focussed on quantitative variables to align with the nature of this review. The identified extracted outcome measures- both subjective and objective- were quantitative outcomes used in more than half of the included studies.

### Subjective outcome measures

#### Numerical pain rating scale (NRS)

The NRS is a widely employed self-report instrument to gauge the intensity of pain experienced by individuals [29]. It uses an 11-point numerical scale, typically ranging from 0 to 10, where 0 indicates “no pain” and 10 signifies “the worst possible pain.” Participants are instructed to select the number that best reflects their current pain level, providing a straightforward and efficient methods for quantifying subjective pain experiences. Young et al. (2010) assessed the NRS for test-retest reliability among subjects with cervical spine pain and found that the scale exhibited a fair reliability, with a minimal detectable change of 4.1 and a threshold of 2.2 minimally clinically important differences (MCID) [29, 30].

#### Headache disability index (HDI)

The HDI is a specialized questionnaire designed to evaluate the impact of headaches on an individual’s daily life and overall functioning [31]. It encompasses questions regarding headache severity, frequency, and how headaches affect various aspects of life, such as work, social activities, and emotional well-being. Widely applied in headache-related research, including studies on migraine and tension-type headaches, the HDI provides valuable insights beyond mere pain intensity, offering a comprehensive understanding of the functional consequences of headaches. Parker et al. (2013) conducted a comprehensive assessment of one-year outcomes and determined a MCID of 13.8% for the HDI [31].

#### Neck disability index (NDI)

The NDI is a self-report questionnaire specifically designed for individuals experiencing neck pain [31]. It assesses the impact of neck pain on daily activities and functioning, covering areas related to pain intensity and its interference with personal care, lifting, reading, work, driving, sleeping, and recreation. Scoring ranges from 0 to 50 points, with 50 indicating the worst level of disability. The MCID is recognised as a 10-point improvement [32]. Commonly used in research on neck pain and cervical spine disorders, the NDI allows researchers to evaluate the degree of disability associated with neck pain and monitor changes in functional status over time. Particularly relevant in studies involving interventions or treatments for neck pain, the NDI serves as a valuable tool for assessing the broader impact beyond pain intensity. The index has demonstrated fair test-retest reliability, further solidifying its role in reliable data collection [31, 32].

### Objective outcome measures

#### Cervical range of motion (CROM)

CROM assessment serves several important purposes in clinical practice and rehabilitation science, including evaluating functional limitations, quantifying impairments, planning and monitoring treatments, identifying cervical spine disorders, and screening for red flags. Range of motion is a widely used parameter [33] for assessing spine movements, yet it can be challenging to measure accurately due to the complex anatomy and associated movement patterns. The CROM is often employed as both a baseline and outcome measure to document the effects of interventions and to adjust treatment plans in clinical practice [34]. Six planes of cervical spine motion are assessed using a CROM goniometer, a clinically valid and reliable tool. These planes include flexion-extension, right and left lateral flexion, and right and left rotation. The reliability and validity of CROM goniometer has been found to be highly reliable for all cervical spine movement with intraclass correlation coefficient (ICC) ranging 0.58–0.99 and presented good validity when compared to the X-ray gold standard with (ICC) ranging 0.82–0.98 [35].

#### Pressure pain threshold (PPT)

The PPT parameter was measured using a handheld algometer (WAGNER PAIN FPK/FPN Algometry unit with the 1 square-centimetre rubber tip application surface). Algometer is an effective method for quantifying PPT. The reliability of this parameter in assessing spinal muscle pain was evaluated by Potter et al. (2006) [36] in a small sample of 10 healthy subjects, demonstrating good within-session reliability with an ICC greater than 0.91 and good between-session reliability with an ICC of greater than 0.87. For this study, if more than one muscle was assessed, only the first reported muscle was included in the analysis.

## Results

Of the 269 dissertations screened, this scoping review included 25 studies conducted at the two universities in SA between 1995 and 2020. Of these, 11 studies were undertaken at UJ [37–47], while 14 were completed at DUT [48–61]. The included studies involved a total of 921 patients and addressed various types of headaches, including cervicogenic (*n* = 6) [37, 39, 40, 47–49], migraine (*n* = 3) [38, 52, 58], and tension-type headaches (*n* = 16) [41, 42, 44–46, 50, 51, 53–57, 59–61]. Comparative randomised clinical trial study designs were adopted in 16 of the studies [37, 39–43, 45, 46, 52–54, 56, 58–61], while experimental randomised clinical trial study designs were used in 6 studies [44, 49–51, 55, 57]. Only one study was a randomised controlled trial [48], and the remaining two were observational studies [38, 47]. Sample sizes varied across studies, ranging from *n* = 20 to *n* = 70 participants, with treatment durations varying from a few days to several weeks and different data collection time points. Further details on the included studies can be found in Table 2.

All 25 studies included a manipulation-only group. These manipulation groups were compared to various other interventions, which included electrotherapeutic modalities, such as electromechanical adjusting instruments (*n* = 1) [49], placebo TENS (*n* = 1) [54], ultrasound therapy (*n* = 2) [39, 40], interferential current (*n* = 2) [40, 53], low-level laser (*n* = 1) [42]. Additionally, dry needling techniques were used, including myofascial dry needling therapy (*n* = 2) [37, 50] and acupuncture points needling (*n* = 2) [44, 58]. Manual therapies included manual traction (*n* = 1) [61], ischemic compression (*n* = 1) [41], soft tissue protocols (*n* = 3) [43, 45, 46]. Medication interventions consisted of Ibuprofen^®^ (*n* = 1) [51], paracetamol (*n* = 1) [55], and paracetamol with caffeine (*n* = 1) [57]. Other interventions included homeopathic migraine complex pills (*n* = 1) [52], cryotherapy (*n* = 1) [59], music therapy (*n* = 1) [60], and nocturnal bite guards (*n* = 1) [56].

Subjective outcome measures included neck pain, headaches, and disability that used a wide variety of self-reported tools across the studies. These included the Neck Disability Index (*n* = 15) [37, 43–45, 47, 49, 51, 52, 55–61], Headache Disability Index (*n* = 11) [37–41, 43, 44, 47–50], Numerical Pain Rating Scale (NRS) (*n* = 15) [39, 40, 42, 44–49, 51, 53, 55–57, 59], and the Headache Diary (*n* = 8) [38, 48, 50, 51, 53–55, 61]. Additionally, subjective measures included the TMJ Symptom Questionnaire (*n* = 1) [41], the Glasgow Homeopathic Hospital Outcome Score (*n* = 1) [52], and a Symptom Diagram (*n* = 1) [60]. Additional objective outcome measures aside from CROM (*n* = 12) [37, 39, 44, 46, 47, 49, 50, 56–58, 60, 61] and PPT (*n* = 9) [40, 42, 43, 45, 46, 50, 53, 55, 57] were Vernier Callipers (*n* = 1) [41], Surface Electromyography (*n* = 1) [48], Biopac-TSD121C Dynamometer (*n* = 1) [48], and the Myofascial Diagnostic Scale (*n* = 1) [53].

### Subjective outcome measures

As shown in Table 3, the mean NRS scores consistently decreased at follow-up for every study, with reductions ranging from 0.12 to 6.39 points across the 16 studies. The most improved were observed with manipulation combined with interferential current intervention [59] while the smallest reduction occurred with the acetaminophen/caffeine intervention without manipulation [57]. In most studies, combining manipulation with an additional modality led to greater improvements compared to manipulation or the comparison group alone, although a study that included cryotherapy was an exception to this trend [59].

**Table 3:** Summary of studies including the numerical pain rating scale (*n* = 16)

Student, Year	Manipulation Plus	No Manipulation	Manipulation Only		Manipulation Plus		No Manipulation	
			Mean Change	*n*	Mean Change	*n*	Mean Change	*n*
Angus, 1997 [58]	Manipulation & Cryotherapy	NA	-0.43	15	-0.29	15	---	---
Donkin, 1998 [60]	Manipulation & Manual Traction	NA	-2.56	15	-2.11	15	---	---
Thomson, 2000 [56]	NA	Acetaminophen 1000 mg / Caffeine 130 mg	-1.49	35	---	---	-0.12	37
Kidson, 2001 [54]	NA	Acetaminophen Acid 500 mg	-0.83	30	---	---	-0.41	30
Cartwright, 2002 [55]	Manipulation & Noctural bite guard	NA	-3.74	15	-4.54	15	---	---
Prithipal, 2003 [52]	Manipulation & Interferential current	Interferential Current	-5.03	20	-6.39	20	-5.66	20
Legoete, 2010 [50]	NA	Ibuprofen	-3.60	16	---	---	-1.70	16
Judelman, 2011 [36]	Manipulation & Myofascial Dry Needling Therapy	Myofascial Dry Needling Therapy	-1.76	16	-2.08	16	-1.25	16
Workman, 2011 [46]	NA	NA	-1.27	30	---	---	---	---
Keshav, 2012 [39]	Manipulation & Interferential Current & Ultrasound Therapy	NA	-5.14	15	-5.99	15	---	---
Orkan, 2012 [43]	NA	Acupuncture Points Needling	-1.00	16	---	---	-1.50	16
Omar, 2015 [41]	Manipulation & Low-level laser therapy	Low-Level Laser Therapy	-4.90	10	-5.30	10	-4.80	10
Seejarim, 2016 [45]	Manipulation & Muscle Energy Technique	Muscle Energy Technique	-4.20	10	-4.70	10	-3.70	10
Dulabh, 2017 [38]	Manipulation & Ultrasound Therapy	NA	-3.33	15	-3.60	15	---	---
Whittaker, 2018 [48]	NA	Electromechanical Adjusting Instrument	-3.42	21	---	---	-3.65	20
Orr, 2018 [44]	Manipulation & MTRT	Muscle Tension Release Treatment (MTRT)	-2.50	10	-4.50	10	-4.40	10
Brann, 2020 [47]	NA	Placebo Control	-1.75	15	---	---	0 -0.50	15 15
*- No manipulation in this group								

^a^ DUT: Durban University of Technology

^b^ UJ: University of Johannesburg

^c^ NA: Not Applicable

Table 4 displays the changes in the mean HDI scores for the 11 studies. These scores ranged from a reduction of 1.97 to 47.74 points. The largest improvement was noted when manipulation was combined with dry needling therapy intervention [50], while the smallest change was observed in a manipulation-only intervention [38]. Generally, the combination of manipulation with another modality resulted in equal or greater improvements compared to manipulation alone or the comparison group. However, an exception was noted in a study that combined manipulation with ultrasound therapy [39].

**Table 4:** Summary of studies including the headache disability index (*n* = 11)

Student, Year	Manipulation Plus	No Manipulation	Manipulation Only		Manipulation Plus		No Manipulation	
			Mean Change	*n*	Mean Change	*n*	Mean Change	*n*
Trollope, 2010 [49]	Manipulation & Myofascial Dry Needling Therapy	Myofascial dry needling therapy	-34.27	15	-47.74	15	-37.73	15
Judelman, 2011 [36]	Manipulation & Myofascial Dry Needling Therapy	Myofascial dry needling therapy	-13.88	16	-13.88	16	-17.75	16
Workman, 2011 [46]	NA	NA	-6.90	30	---	---	---	---
Keshav, 2012 [39]	Manipulation & Interferential Current & Ultrasound Therapy	NA	-21.47	15	-25.73	15	---	---
Orkan, 2012 [43]	NA	Acupuncture Points Needling	-11.18	16	---	---	-14.38	16
Moosajee, 2013 [40]	Manipulation & Ischemic Compression	Ischemic Compression	-20.00	16	-29.88	16	-28.13	16
Chopdat, 2015 [37]	NA	NA	-1.97	20	---	---	---	---
Newman, 2015 [42]	Manipulation & Soft Tissue Protocol Combination	Soft Tissue Protocol / Massage	-22.20	10	-23.00	10	-14.00	10
Dulabh, 2017 [38]	Manipulation & Ultrasound Therapy	NA	-35.80	15	-34.00	15	---	---
Whittaker, 2018 [48]	NA	Electromechanical Adjusting Instrument	-27.33	21	---	---	3.65	20
Brann, 2020 [47]	NA	Placebo Control	-10.00	15	---	---	-4.00 -2.00	15 15
*- No manipulation in this group								

The NDI scores are presented in Table 5 for the 15 studies, all of which showed a reduction in mean NDI scores at follow-up, with decreases 0.2 to 23.0 points. The largest change was observed with the intervention combining manipulation and soft tissue work [47] while the smallest change occurred in the acetaminophen/caffeine intervention [57]. Overall, there was no consistent pattern indicating larger changes for any specific intervention or combination of interventions.

**Table 5:** Summary of studies including the neck disability index (*n* = 15)

Student, Year	Group (with manipulation)	Group (without manipulation)	Manipulation		Group (with manipulation)		Group (without manipulation)	
			Mean Change	*n*	Mean Change	*n*	Mean Change	*n*
Da Silva, 1994 [59]	Manipulation & Music Therapy	NA	-13.20	15	-14.47	15	---	---
Angus, 1997 [58]	Manipulation & Cryotherapy	NA	-17.33	15	-14.80	15	---	---
Donkin, 1998 [60]	Manipulation & Manual Traction	NA	-17.33	15	-10.94	15	---	---
Cullinan, 1998 [57]	Manipulation & Acupunture	NA	-9.20	15	-1.70	15	---	---
Thomson, 2000 [56]	NA	Acetaminophen 1000 mg / Caffeine 130 mg	-2.82	35	---	---	-0.20	35
Kidson, 2001 [54]	NA	Acetylsalicylic Acid 500 mg	-3.97	30	---	---	-2.33	30
Cartwright, 2002 [55]	Manipulation & Occlusion Splint Therapy	NA	-3.33	15	-3.73	15	---	---
du Preez, 2004 [51]	Manipulation & Homeopathic Migraine Pills Combination	Homeopathic Migraine Pills	-17.45	10	-17.78	10	-18.50	10
Legoete, 2010 [50]	NA	Ibuprofen	-7.00	16	---	---	-6.40	16
Judelman, 2011 [36]	Manipulation & Myofascial Dry Needling Therapy	Myofascial Dry Needling Therapy	-10.13	16	-14.25	16	-10.62	16
Workman, 2011 [46]	Manipulation & Soft Tissue Protocol	NA	-6.90	30	-23.00	10	---	---
Orkan, 2012 [43]	NA	Acupuncture Points Needling	-11.18	16	---	---	-14.38	16
Newman, 2015 [42]	NA	Soft Tissue Protocol	-22.20	10	---	---	-14.00	10
Orr, 2018 [44]	Manipulation & MTRT	Muscle Tension Release Treatment (MTRT)	-18.00	10	-13.00	10	-10.00	10
Whittaker, 2018 [48]	NA	Electromechanical Adjusting Instrument	-9.05	21	---	---	-11.30	20

^a^ DUT: Durban University of Technology

^b^ UJ: University of Johannesburg

^c^ NA: Not Applicable

### Objective outcome measures

Table 6 presents data from 12 studies that show both increases and decreases in CROM at follow-up across all planes; however, the majority of interventions led to an increase in CROM. For flexion, changes ranged from a decrease of -4.5 degrees (manipulation combined with manual traction) [61] to an increase of 11.7 degrees (electromechanical adjusting instrument) [49]. In extension, changes varied from − 1.7 degrees (manipulation and acupuncture [58]; to 8.9 degrees (manipulation) (42). For right rotation, the range was − 2.3 degrees (acetaminophen/caffeine) [57] to 12.1 degrees (manipulation) [38]. Left rotation changes ranged from − 1.9 degrees (acetaminophen/caffeine) [57] to 13.6 degrees (manipulation) (38). In right lateral flexion, changes varied from − 2.0 degrees (manipulation) (43) to 10.3 degrees (manipulation) [38], and in left lateral flexion, the range was − 2.5 degrees (manipulation and manual traction) [61] to 11.3 degrees (Muscle Energy Technique) [37].

**Table 6:** Summary of studies including the cervical range of motion (*n* = 12)

Angus (1997) [58]	Manipulation	-1.30	15	-0.17	15	2.13	15	0.30	15	0.07	15	0	15
	Manipulation & Cryotherapy	-3.84	15	2.00	15	3.07	15	1.30	15	0.13	15	1.20	15
Cullinan (1998) [57]	Manipulation	-0.30	15	4.20	15	7.20	15	4.60	15	-2.00	15	0.60	15
	Manipulation & Acupunture	1.20	15	-1.70	15	6.60	15	0.40	15	0.30	15	0.70	15
Donkin (1998) [60]	Manipulation	2.40	15	5.90	15	1.30	15	0.90	15	2.20	15	4.30	15
	Manipulation & Manual Traction	-4.50	15	1.30	15	-0.20	15	1.30	15	1.00	15	-2.50	15
Thomson (2000) [56]	Manipulation	9.43	35	6.77	35	11.17	35	12.69	35	10.34	35	10.89	35
	Acetaminophen 1000 mg / Caffeine 130 mg	-0.68	35	0.11	35	-2.29	35	-1.91	35	-0.18	35	-0.72	35
Cartwright (2002) [55]	Manipulation	2.54	15	-0.66	15	4.66	15	14.4	15	0.66	15	5.14	15
	Manipulation & Nocturnal bite guard	3.66	15	0.34	15	7.40	15	12.00	15	4.06	15	3.54	15
Trollope (2010) [49]	Manipulation	7.73	15	8.94	15	12.13	15	13.6.	15	9.20	15	8.13	15
	Myofascial Dry Needling Therapy	7.87	15	3.87	15	9.13	15	6.40	15	6.93	15	4.94	15
	Manipulation & Myofascial Dry Needling Therapy	4.54	15	3.74	15	5.34	15	9.53	15	3.74	15	4.80	15
Judelman (2011) [36]	Manipulation	7.38	16	6.69	16	3.32	16	4.37	16	7.12	16	6.00	16
	Myofascial Dry Needling Therapy	5.56	16	2.94	16	5.56	16	4.50	16	6.75	16	4.44	16
	Manipulation & Myofascial Dry Needling Therapy	3.75	16	6.25	16	6.50	16	6.25	16	7.75	16	7.31	16
Workman (2011) [46]	Manipulation	3.58	30	5.05	30	4.88	30	4.82	30	1.62	30	1.58	30
Orkan (2012) [43]	Manipulation	-2.49	16	7.81	16	3.94	16	1.18	16	2.81	16	5.25	16
	Acupuncture Points Needling	-0.19	16	4.25	16	2.62	16	3.31	16	1.00	16	0.06	16
Seejarim (2016) [45]	Manipulation	6.80	10	8.20	10	7.00	10	8.40	10	6.40	10	6.20	10
	Muscle Energy Technique	10.60	10	6.80	10	6.70	10	5.30	10	6.80	10	11.30	10
	Manipulation & Muscle Energy Technique	4.30	10	5.20	10	4.70	10	3.60	10	8.50	10	8.60	10
Dulabh (2017) [38]	Manipulation	1.67	15	3.06	15	2.73	15	1.73	15	2.20	15	2.20	15
	Manipulation & Ultrasound Therapy	1.74	15	3.14	15	2.40	15	1.60	15	3.07	15	2.73	15
Whittaker (2018) [48]	Manipulation	10.05	21	5.05	21	9.00	21	9.81	21	7.29	21	7.95	21
	Electromechanical Adjusting Instrument	11.70	20	6.40	20	6.30	20	4.60	20	5.60	20	3.75	20

^a^ DUT: Durban University of Technology

^b^ UJ: University of Johannesburg

^c^ NA: Not Applicable

Table 7 shows an increased mean PPT score at follow-up for all interventions included in the analysis of the 9 studies, except for a decrease of -0.16 [57]. Changes in scores ranged between 0 for an acetaminophen intervention [55] and 4.8 for a manipulation-only intervention [45]. No clear trend emerged to suggest a pattern among the different groups.

**Table 7:** Summary of studies including the pressure pain threshold (*n* = 9)

			Manipulation		Group (with manipulation)		Group (without manipulation)	
Student, Year	Group (with manipulation)	Group (without manipulation)	Mean Change	*n*	Mean Change	*n*	Mean Change	*n*
Thomson, 2000 [56]	NA	Acetaminophen 1000 mg / Caffeine 130 mg	-0.16	35	---	---	0.61	35
Kidson, 2001 [54]	NA	Acetaminophen Acid 500 mg	0.12	30	---	---	0	30
Prithipal, 2003 [52]	Manipulation & Interferential current	Interferential Current	0.2	20	0.3	20	0.4	20
Trollope, 2010 [49]	Manipulation & Myofascial Dry Needling Therapy	Myofascial dry needling therapy	0.79	15	1.08	15	1.28	15
Orr, 2018 [44]	Manipulation & MTRT	Muscle Tension Release Treatment (MTRT)	4.8	10	4.1	10	2.8	10
Keshav, 2012 [39]	Manipulation & Interferential Current & Ultrasound Therapy	NA	1.08	15	1.18	15	---	---
Newman, 2015 [42]	Manipulation & Soft Tissue Protocol Combination	Soft Tissue Protocol / Massage	1.9	10	1.89	10	2.1	10
Omar, 2015 [41]	Manipulation & Low-level laser therapy	Low-Level Laser Therapy	0.83	10	0.71	10	0.99	10
Seejarim, 2016 [45]	Manipulation & Muscle Energy Technique	Muscle Energy Technique	0.75	10	0.93	10	1.02	10

^a^ DUT: Durban University of Technology

^b^ UJ: University of Johannesburg

^c^ NA: Not Applicable

## Discussion

Conducting high-quality original research in all regions of the world is highly complicated and expensive and truly not feasible. This study explored readily available Master’s dissertations, a source of grey literature, that conducted clinical trials using spinal manipulative therapy (SMT) for headache patients seeking care at clinics associated with two SA chiropractic programs. The findings from this review align with existing higher-quality published clinical trials (6,47–50) and leading guidelines (10,51). Specifically, SMT, along with other non-pharmacological modalities, improves both subjective and objective outcome measures. This reinforces the present study’s findings that SMT, along with other care options within the scope of chiropractic practice, has a positive effect on primary headaches. It highlights the importance of further exploring the chiropractic profession for headache management in SA.

In SA, significant socioeconomic and healthcare disparities persist, compounded by poverty, unemployment, and a high burden of disease that limits access to care [62]. These challenges are particularly acute for those reliant on the public healthcare system, where chiropractic services are not included [63]. In response, SA is working toward establishing a national health system that ensures equal access to health care for all [62].

Grey literature can play a crucial role in bridging the gap between effective and accessible headache management by providing relevant data on local communities that typically lack access to private chiropractic care and are underrepresented in traditional research [63–65]. This information can inform policymakers to better understand the potential value of care that is not currently included in their health system, such as chiropractic, highlighting its role in managing conditions like headaches and contributing to broader health outcomes [66, 67]. By offering insights into underserved populations, this grey literature can support health equity initiatives to align with both the Sustainable Development Goals (SDGs) for good health and well-being and South Africa’s National Development Plan (NDP) 2030, which emphasizes improving healthcare access, quality, and equity with reduction in the burden of non-communicable diseases including primary headaches, with evidence-based interventions [6, 68].

This study emphasizes that in the absence of published literature for patients in specific regions, such as SA, grey literature from reputable sources can serve as a valuable resource. To ensure the credibility of the information, this study verified that the data collection approaches taken by the SA institutions were similar or better than those for funded clinical trials. A significant advantage of using Masters dissertations was the standardization of outcome measurements across studies and institutions, allowing for the pooling of data—a feature often lacking in published literature—which strengthens the findings [69]. Furthermore, incorporating grey literature into evidence-based decision-making is valuable, as it ensures that unpublished work, including studies with negative outcomes, is appropriately disseminated [70]. Notably, the studies in this review revealed a consistent trend of positive outcomes.

A common limitation with the use of grey literature in evidence-based discussions is similar to that associated with observation studies: effect size can be impacted by the inherent methodological weaknesses. Many of these weaknesses arise from the absence of outlined processes, such as the peer review process typically present in published literature. However, for many grey literature products, including the theses included in this review, there are peer-review processes that may be more rigorous than many journals due to the standardization within the graduation procedures [71].

A key limitation of this review is the sample size of the included studies, which can directly affect the observed effect size [72]. Although larger sample sizes are sometimes viewed as complicating the interpretation of statistical significance and clinical meaningfulness [72], as well as increasing costs, establishing and meeting sample size estimations is critical to ensure both ethical and impactful research is done. Despite the small sample sizes of the individual studies, each offers valuable insights that collectively strengthen the overall understanding of the topic, cumulatively they provide a broader perspective that enhances the evidence base.

Another limitation specific to this review is the inconsistent reporting of standard deviations in the included studies. This inconsistency hampers the ability to synthesize the data and draw more meaningful interpretations [73]. The involved institutions adapt their data analysis protocols and marking criteria to ensure consistent reporting of standard deviations, as this would substantially enhance the value of future study synthesis and discussions regarding study results in comparison to minimal clinically important differences (MCID). An additional limitation was our limited ability to further stratify the outcome measures based on overall significance, clinical relevance, or other pertinent factors. This constraint reduced the granularity of the analysis and may have hindered deeper insights into the varying importance of specific outcomes.

One final limitation is that research conducted by students may be more susceptible to errors, which can diminish the quality of the data and study outcomes [74]. However, it is important to note that all the dissertations included in this review employed validated assessment tools and followed standardized protocols, providing a degree of methodological rigor. Similar to the standard deviation limitation, we encourage the institutions involved to address the potential for selection bias inherent in these environments to be addressed within the student’s study protocol, actively monitored throughout data collection, and carefully considered its implication when interpreting and synthesizing results.

To the best of the authors’ knowledge, this is the first scoping review of grey literature on chiropractic institutional Master’s dissertations. This review highlights the value of such work while also acknowledging the current limitations in project data collection for this purpose. While evidence suggests that including grey literature can minimize the overestimation of treatment effects, quantifying the true value of this type of study remains challenging [75]. Nevertheless, given the limited resources available to inform policymakers in SA, this review provides a foundational evidence base to explore the chiropractic profession’s management of headache patients within the SA region.

## Conclusion

This grey literature scoping review identified 25 chiropractic Master’s dissertations that evaluated the use of chiropractic spinal manipulative therapy (SMT) for 921 headache patients in South Africa (SA). The subjective and objective outcomes were consistent with those reported in published clinical trials and guidelines, indicating that SMT combined with other non-pharmacological treatments yield the improvements when compared to other treatment options without SMT. The grey literature reviewed in this study can be a valuable tool for addressing existing healthcare disparities in South Africa by providing insights into populations lacking access to chiropractic care in the public health system. This research can inform policy decisions to consider the integration of chiropractic services into public health care to align with the National Development Plan (NDP) 2030 and Sustainable Development Goals (SDGs) to enhance musculoskeletal healthcare access, ultimately advancing universal health coverage and better patient outcomes. Despite the inherent limitations of grey literature, these findings are valuable for healthcare decision-making, particularly in resource-limited settings. This is especially relevant in regions like sub-Saharan Africa, where data are scarce but effective treatments are urgently needed.

## Data Availability

All data generated or analysed during this study are included in this published article. Raw data are stored in the Parker University Research Repository (accessed via https://my.parker.edu/ICS/Research/Research_Data_Repository.jnzfor researchers who meet the criteria for access to this material.
